# Ling’s Adsorption Theory as a Mechanism of Membrane Potential Generation Observed in Both Living and Nonliving Systems

**DOI:** 10.3390/membranes6010011

**Published:** 2016-01-26

**Authors:** Hirohisa Tamagawa, Makoto Funatani, Kota Ikeda

**Affiliations:** 1 Department of Human and Information Systems, Faculty of Engineering, Gifu University, 1-1 Yanagido, Gifu, Gifu 501-1193, Japan; ship_ship19931125@icloud.com; 2 Graduate School of Advanced Mathematical Sciences, Meiji University, 4-21-1, Nakano, Nakano-ku, Tokyo 165-8525, Japan; ikeda@meiji.ac.jp

**Keywords:** membrane potential, action potential, ion permeation, Goldman-Hodgkin-Katz, ion adsorption, Ling’s adsorption theory

## Abstract

The potential between two electrolytic solutions separated by a membrane impermeable to ions was measured and the generation mechanism of potential measured was investigated. From the physiological point of view, a nonzero membrane potential or action potential cannot be observed across the impermeable membrane. However, a nonzero membrane potential including action potential-like potential was clearly observed. Those observations gave rise to a doubt concerning the validity of currently accepted generation mechanism of membrane potential and action potential of cell. As an alternative theory, we found that the long-forgotten Ling’s adsorption theory was the most plausible theory. Ling’s adsorption theory suggests that the membrane potential and action potential of a living cell is due to the adsorption of mobile ions onto the adsorption site of cell, and this theory is applicable even to nonliving (or non-biological) system as well as living system. Through this paper, the authors emphasize that it is necessary to reconsider the validity of current membrane theory and also would like to urge the readers to pay keen attention to the Ling’s adsorption theory which has for long years been forgotten in the history of physiology.

## 1. Introduction

A cell generates nonzero potential across the plasma membrane, which is called membrane potential [1,2,3,4,5,6,7,8,9,10,11,12,13,14,15,16]. Cell exhibits vehement change of membrane potential in the active state, and such a potential is called action potential. According to the Goldman-Hodgkin-Katz equation (GHK equation), membrane potential is generated by the mobile ion passage through the plasma membrane [1,7,17,18]. Membrane potential is determined by the ion concentration inside and outside of cell and the ion flux through the plasma membrane. It is broadly acknowledged that the GHK equation precisely predicts the actual membrane potential behavior. This successful outcome is interpreted as the ion passage through the plasma membrane is actually one of the fundamental factors for the nonzero membrane potential generation of the cell. However, nonzero potential is even generated across an artificial impermeable membrane separating two electrolytic solutions [19,20,21,22]. Colacicco’s report is a typical example [19,20]. Colacicco observed the nonzero potential generation across oil membrane impermeable to ions.

Tamagawa, one of authors of this paper, and Morita also reported that the membrane potential across the membrane impermeable to ions was virtually identical to the membrane potential across a permeable membrane, and the those experimentally obtained membrane potentials were identical to the membrane potential theoretically obtained by the use of GHK equation [21,22]. Tamagawa and Morita basically dispute the currently accepted mechanism of membrane potential generation of cell. They take side with the adsorption theory advocated by Dr. Gilbert Ling [1,7,21,22]. Ling has emphasized that the membrane potential of cell is basically caused by the adsorption of mobile ions onto the membrane adsorption sites not by the ion permeation through the membrane. The Ling’s adsorption theory is applicable to the nonliving system as well as living system. In fact, Ling carried out some experiments many years ago, and confirmed that his theory is applicable to the nonliving system [1].

The authors would like to briefly comment on Dr. Ling as a scientist and his accomplishments a little, since the authors employ his theory in this paper. Ling made a historical accomplishment in his young days. He perfected a microelectrode technique, which has greatly advanced the electrophysiology, when he was a Ph.D. candidate in University of Chicago. Later, he advocated his own theory of cell metabolic activity. Since his own theory was completely in conflict with the existing theory, his fame once established in his young days has been declining gradually, and now he is completely left out of science world. Nowadays, nobody knows his accomplishments or people who know his accomplishments think that what he has advocated is a silly talk. However, the authors of this paper do not care about the label put on him and his accomplishments, and the authors employ his theory throughout this work. The authors had to add some more comments as follows: Basically the same idea as the Ling’s adsorption theory was put forth by the late K. L. Cheng independently of Ling, though decades after Ling [23,24,25,26,27]. Cheng was an electrochemist and had studied the electrode mechanism. He reached a conclusion that the potential measured by the electrode is caused by the ion adsorption onto the electrode surface adsorption sites.

Existing theory of cell biology attributes the action potential induction to the function of ion channels and pumps embedded in the plasma membrane [1,4,5,7,13,14,15,16]. Hence, the induction of action potential is one of manifestations of biological activity. Therefore, it is quite natural to believe that the action potential is not observed in the artificial system having neither ion channels nor pumps. Contrary to such a quite natural thought, action potential-like potential has been repeatedly observed in the artificial systems in which no ion channels or pumps were embedded [28,29,30,31,32,33,34,35,36,37,38,39,40,41,42,43,44,45,46,47,48,49,50].

Proteinoid is a tiny sphere consisting of amino acids. It was first created by Sidney Fox [31,32,34,35,40]. Scientists regard it as a protocell or something similar to the protocell. Although the proteinoid was artificially created and it did not contain any ion channel or pump, it generated action potential-like potential [31,32,34,35,40]. Yoshikawa *et al.* reported a number of even more intriguing observations. They separated two electrolytic aqueous solutions with an artificially created liquid membrane. The experimental systems they used did not contain any biological material unlike Fox's. However, Yoshikawa *et al.* observed continuous generation of action potential-like potential [33,34,37,38,39,41,42], and other research groups also observed similar phenomena [43,44,45,46,47,48,49]. Yoshikawa *et al.* did not seem to have so strong interest in pursuing the physiological meaning of their observations. Their interest seemed to primarily lie in the physical and mathematical analysis of their observations. We think that those observations by Yoshikawa *et al.* imply a grave physiological meaning. That is, generation of membrane potential including action potential may not be a characteristic particular only to living system, and no ion channel or pumps are needed for generating action potential. The membrane potential generation of cell may not represent the biological activity, and it may be merely a consequence of physical chemistry-based nature of electrolytic solution systems. *i.e.*, generation of membrane potential including action potential has nothing to do with life and death. According the GHK equation, ion transfer through the plasma membrane is essential for the membrane potential and action potential generation [1,7,17,18], but it might be possible to generate them without ion transfer through the membrane. Therefore, in this work, the authors attempted to achieve membrane potential generation and action potential-like potential generation between two electrolytic solutions without ion transfer through the membrane, then the authors discussed the physiological implication of the experimental observations.

## 2. Preparation of Membranes and Solutions

The authors made measurements of potential between two electrolytic solutions separated by an impermeable material. Those potential measurements were categorized into two measurements. Exp. I: Measurement of potential between two electrolytic solutions separated by an impermeable membrane and Exp. II: Measurement of oscillating potential between two electrolytic solutions separated by a silver wire & liquid membrane, where the silver wire and liquid membrane is of course impermeable to ions.

### 2.1. Fabrication of Impermeable Membrane

Two types of impermeable solid membranes were employed for carrying out Exp. I. One was a thin urethane film, MG90 (25 µm thick), manufactured by Takeda Sangyo Co., Ltd. (Tokyo, Japan).

**Figure 1:**
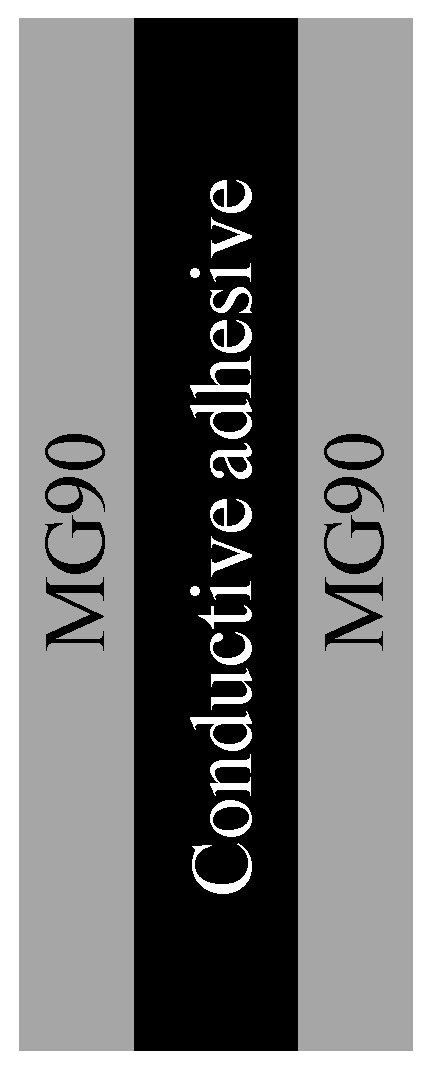
Side view of KBMG90.

Another membrane was fabricated by attaching two sheets of MG90 to each other with an electrically conductive adhesive. The electrically conductive adhesive was prepared simply by mixing carbon (Ketjen black) and epoxy. This membrane fabricated is hereafter called KBMG90. The structure of KBMG90 is shown in Figure 1.

### 2.2. Liquid Membrane

Two types of liquid membranes were prepared for carrying out Exp. I and II. One liquid membrane was prepared by mixing 0.086 g of surfactant SDS with 24 g 1-pentanol. This liquid membrane is hereafter called S-penta. S-penta looked saturated with SDS.

Another one was prepared by mixing 0.11 g of surfactant CTAB with 24 g 1-pentanol. This is hereafter called C-penta.

### 2.3. Electrolytic Solutions

Various concentrations of KCl solution were prepared simply by dissolving KCl into deionized water, and lower concentration of KCl solutions were prepared by diluting the high concentration KCl solution with deionized water. These KCl solution were used for both Exp. I and II. Concentration of those KCl solutions ranged from 0.00001 to 3.4 M.

For Exp. II, two kinds of NaCl aqueous solutions were prepared. One was 0.3 M NaCl aqueous solution, which was prepared by dissolving NaCl into deionized water. Another one was the mixture of 0.3 M NaCl & 1 M ethanol aqueous solution, and it was prepared simply by dissolving NaCl and ethanol into the deionized water.

## 3. Potential Measurement

### 3.1. Exp. I

**Figure 2:**
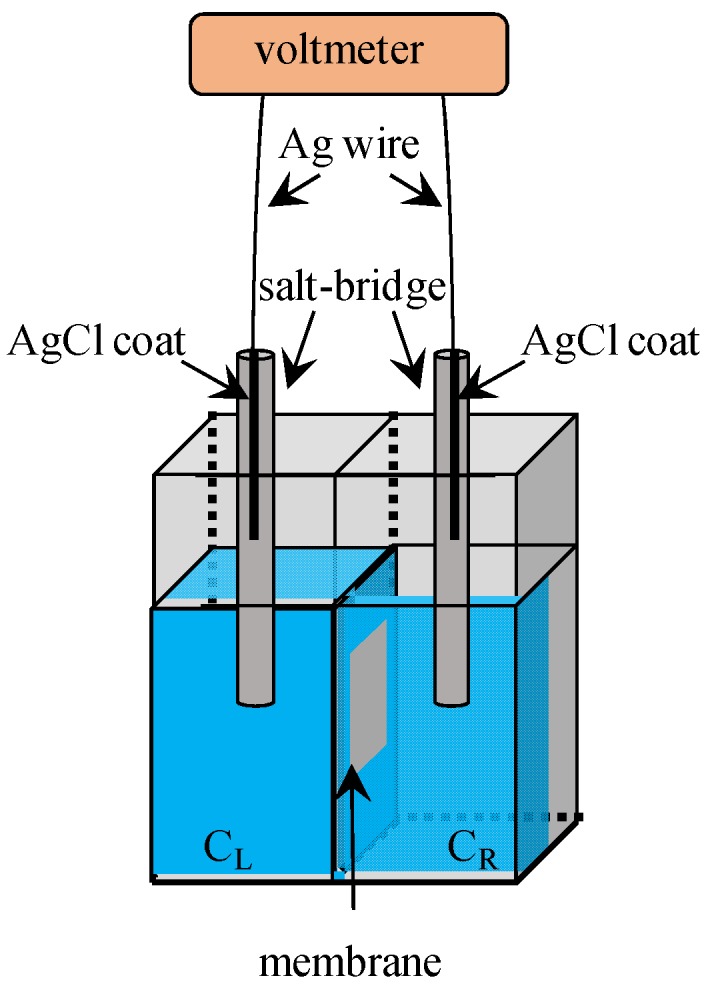
Experimental setup for measuring the potential across a membrane. C_L_ and C_R_ represent the KCl concentration of left and right KCl solutions, respectively.

Figure 2 illustrates the setup for the measurement of potential across a membrane of MG90 coated with a liquid membrane. Two Ag wires served as Ag/AgCl electrode, and their ends were coated with AgCl. Two KCl solutions were supplied in the left and right compartments, respectively, where these KCl concentrations were denoted by C_L_ and C_R_, respectively. Voltmeter shows the stable potential within a few minutes, and that potential was registered.

Another experiment was carried out by using the KBMG90 in place of MG90 coated with a liquid membrane. Procedure of making a measurement was same as when MG 90 was used, but it took longer for the potential to stabilize.

### 3.2. Exp. II

**Figure 3:**
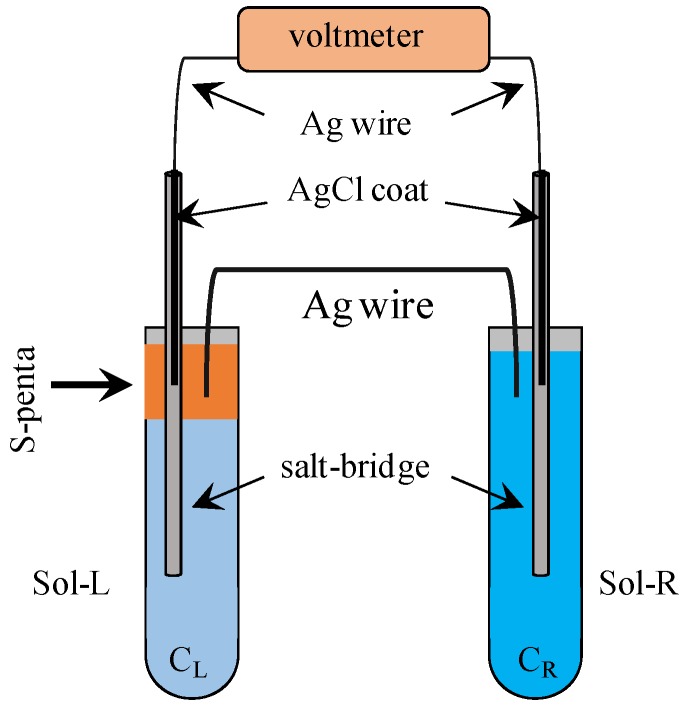
Experimental setup for measuring the potential across the membrane of a silver wire & S-penta. C_L_ and C_R_ represent the concentration of left NaCl and right KCl solutions, respectively. The end of Ag wire which was connected to the voltmeter was coated with AgCl.

Figure 3 illustrates the setup for the measurement of potential across the membrane—a silver wire & S-penta—. NaCl and KCl solutions were supplied in the left and right glass tubes, respectively. Left and right solutions were denoted by Sol-L and Sol-R, respectively. Their KCl concentrations were denoted by C_L_ and C_R_, respectively. Two Ag wires connected to the voltmeter served as an Ag/AgCl electrode, and their ends were coated with AgCl. Voltmeter showed the stable potential within a few minutes after completing the preparation of potential measurement. That potential was registered. Sol-L and Sol-R were electrically connected with the membrane of a silver wire & S-penta. However, ion transfer never took place between Sol-L and Sol-R. Another experiment was carried out by using the C-penta in place of S-penta.

## 4. Results and Discussion

### 4.1. Potential Measurement

#### 4.1.1. Exp. I

**Figure 4:**
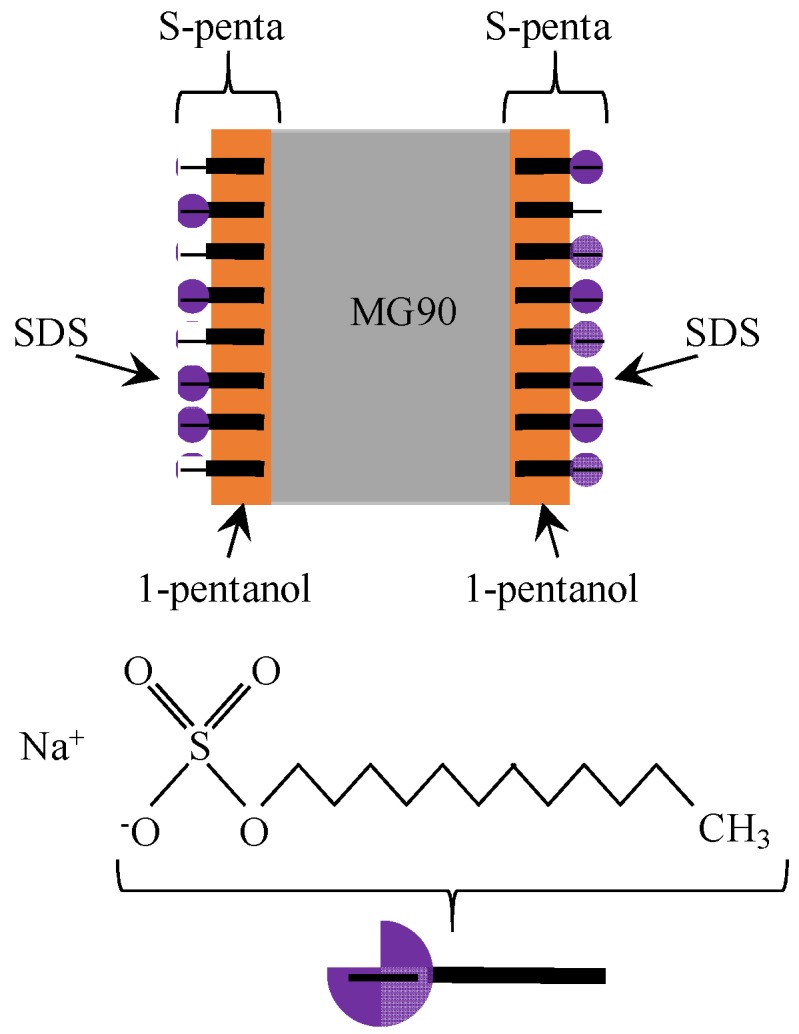
Side view of SMG90 and the structure of SDS.

**Figure 5:**
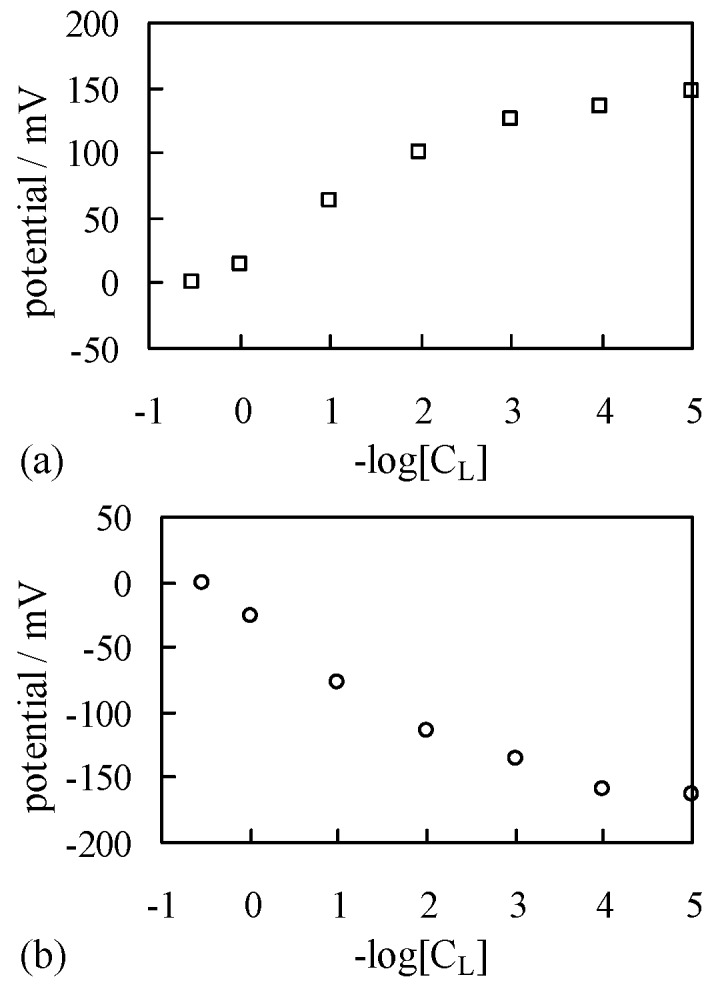
Membrane potential generated across (**a**) SMG90 and (**b**) CMG90 against –log[C_L_].

The potential measurement described in the Section 3.1 was carried out. Membrane preparation should be finalized right before starting the measurement by the following procedure: A few drops of the liquid membrane of S-penta was spread on both surfaces of a MG90, resulting in a MG90 coated with S-penta, and hereafter the resultant membrane is called SMG90. Side view of SMG90 is illustrated in Figure 4. Both surfaces were covered with negatively charged planes, since head group of SDS was negatively charged in the dissociated state. A SMG90 was mounted to the setup in Figure 2. C_R_ was maintained constant at 3.4 M for all the potential measurements. Potential in the left compartment in reference to the right compartment was measured for various C_L_. Figure 5a shows potential *vs.* –log[C_L_]. Potential increases with the decrease of C_L_. Another potential measurement was carried out using another membrane. Another membrane used was the MG90 coated with C-penta. Hereafter, the resultant membrane is called CMG90. Both surfaces were coated with the positively charged planes, since head group of CTAB was positively charged in the dissociated state. Potential measurement was carried out by following the same procedure as described above. Figure 5b shows potential *vs.* –log[C_L_]. Potential decreases with the decrease of C_L_. It is opposite potential behavior to Figure 5a.

Both SMG90 and CMG90 are impermeable to ions. However, nonzero potential across the membrane (membrane potential) was observed. It was in conflict with the basic concept of membrane potential generation mechanism [1,2,3,4,5,6,7,8,9,10,11,12,13,14,15,16,17,18,19,20,21,22]. More concretely, it is widely acknowledged that nonzero membrane potential is generated due to the passage of ions through the membrane according to the GHK equation [1,7]. However, nonzero potential across the impermeable membrane was actually generated as shown in Figure 5. Some people may argue that both SMG90 and CMG90 had a number of pinholes which were invisibly small but large enough for ion passage. Hence, the authors carried out further potential measurements in order to eliminate such a doubt.

**Figure 6:**
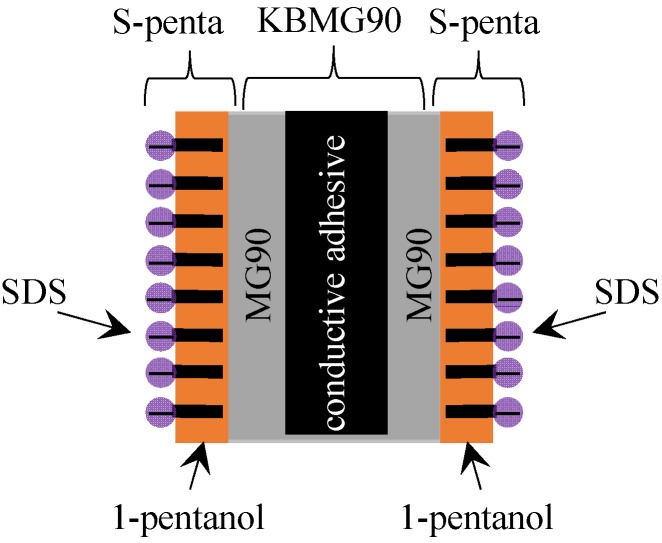
Side view of SKBMG90 coated with S-penta.

The same potential measurement was carried out by the use of a membrane KBMG90, which was completely impermeable to the ions, in place of SMG90 and CMG90. Both surfaces of KBMG90 were coated with S-penta, and the resultant membrane was denoted by SKBMG90. The structure of SKBMG90 is illustrated in Figure 6. Both surfaces were covered with negatively charged planes.

**Figure 7:**
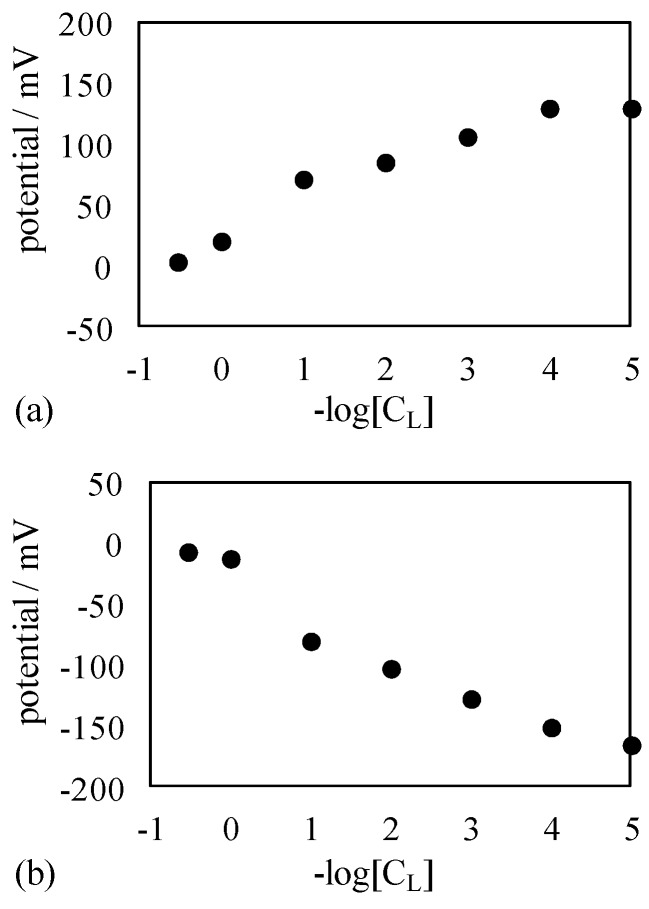
Membrane potential generated across (**a**) SKBMG90 and (**b**) CKBMG90 against –log[C_L_].

Another potential measurement was carried out using another membrane. The membrane used was the KBMG90 coated with C-penta in place of S-penta. Hereafter the resultant membrane is called CKBMG90. Both surfaces were covered with the positively charged planes. Measured potential profiles are shown in Figure 7. The potential data shown in Figure 7 is virtually quantitatively same as that in Figure 5. Hence, it is concluded that even the use of impermeable membrane induces the nonzero membrane potential against the prediction by the existing theory of membrane potential generation mechanism.

#### 4.1.2. Exp. II

**Figure 8:**
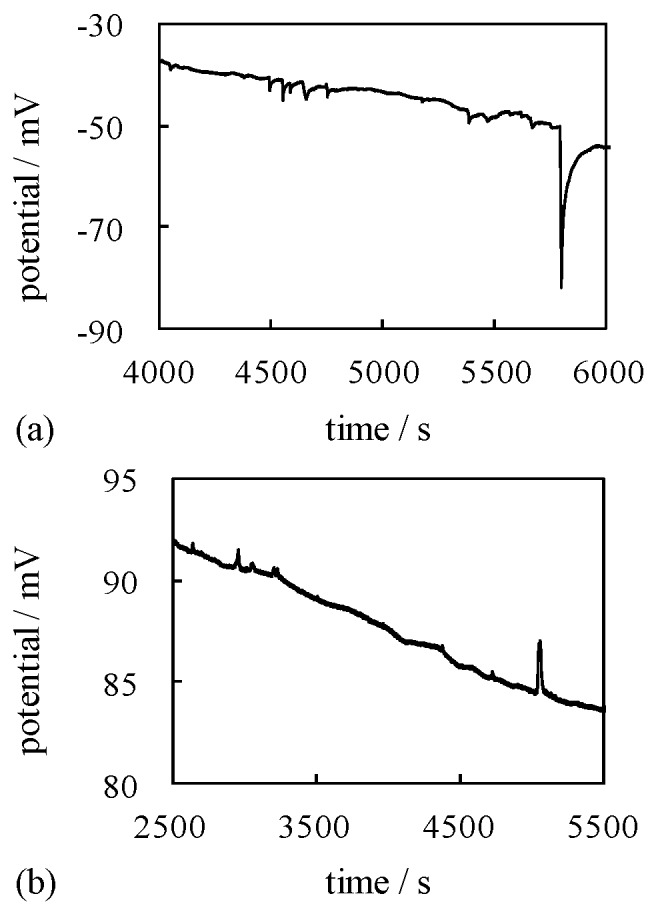
Membrane potential generated across (**a**) a silver wire & S-penta and (**b**) a silver wire & C-penta.

Potential measurement described in the Section 3.2 was carried out. The potential between 0.3 M NaCl and 0.5 M KCl solutions separated by a silver wire & S-penta was measured as a function of time. Spontaneous potential oscillation was observed as shown in Figure 8a.

Potential oscillation between various two electrolytic solutions has been observed and analyzed by a number of research groups up to date [33,36,37,38,39,41,42,43,44,45,46,47,48,49]. Although the oscillation mechanism has not been fully elucidated yet, the occurrence of potential oscillation sometimes is attributed to the transmembrane ion transfer between two electrolytic solutions just like indicated by the concept of GHK equation [33,36,37,38,39,41,42,43,44,45,46,47,48,49]. If not fully attributed to the ion transfer, it has been believed that the ion transfer between two electrolytic solutions plays a role of potential oscillation induction to some extent. Our experimental setup illustrated in Figure 3 never allowed ion transfer, but potential oscillation was induced. Use of a silver wire & C-penta as a separator intervening 0.3 M NaCl and 0.5 M KCl solutions also resulted in the spontaneous induction of potential oscillation as shown in Figure 8b.

**Figure 9:**
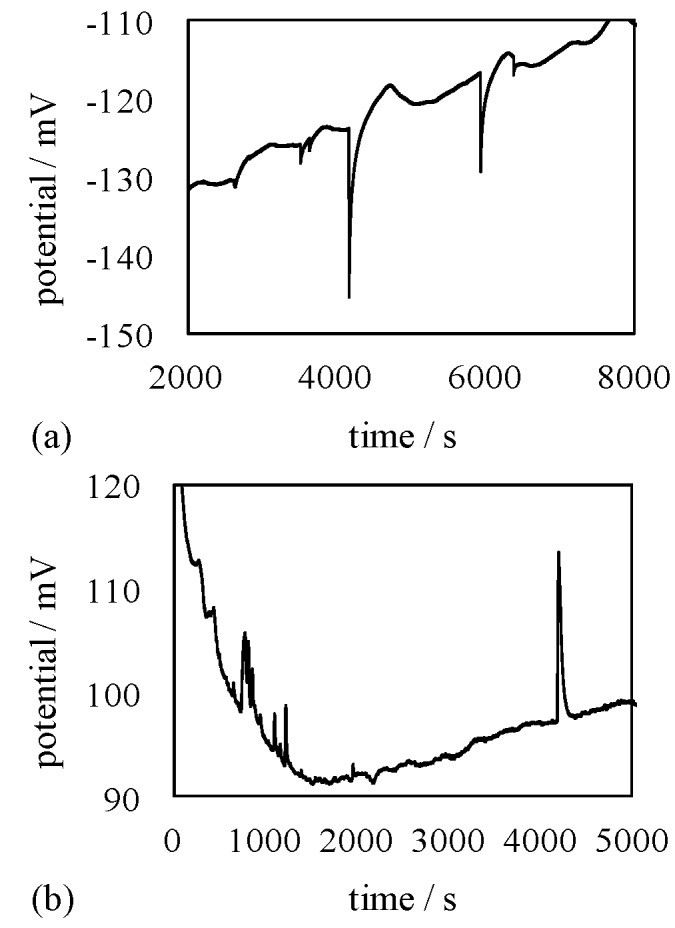
Membrane potential generated across (**a**) a silver wire & S-penta and (**b**) a silver wire & C-penta.

Potential oscillation became more obvious by the use of the new solution—mixture of NaCl, EtOH and deionized water—in place of 0.3 M NaCl, where the concentration of NaCl and EtOH in the new solution was 0.3 M and 1 M, respectively. Potential *vs.* time between the new solution and 0.5 M KCl across the separator of a silver wire & S-penta and of a silver wire & C-penta are shown in Figure 9a,b, respectively. As stated, the oscillation became more clearly induced. We noticed in Figure 8 and Figure 9 that the potential peaks were negative going, when a silver wire & S-penta was employed, while the potential peaks were positive going, when a silver wire & C-penta was employed.

### 4.2. Mechanism of Potential Generation across the Impermeable Membrane

**Figure 10:**
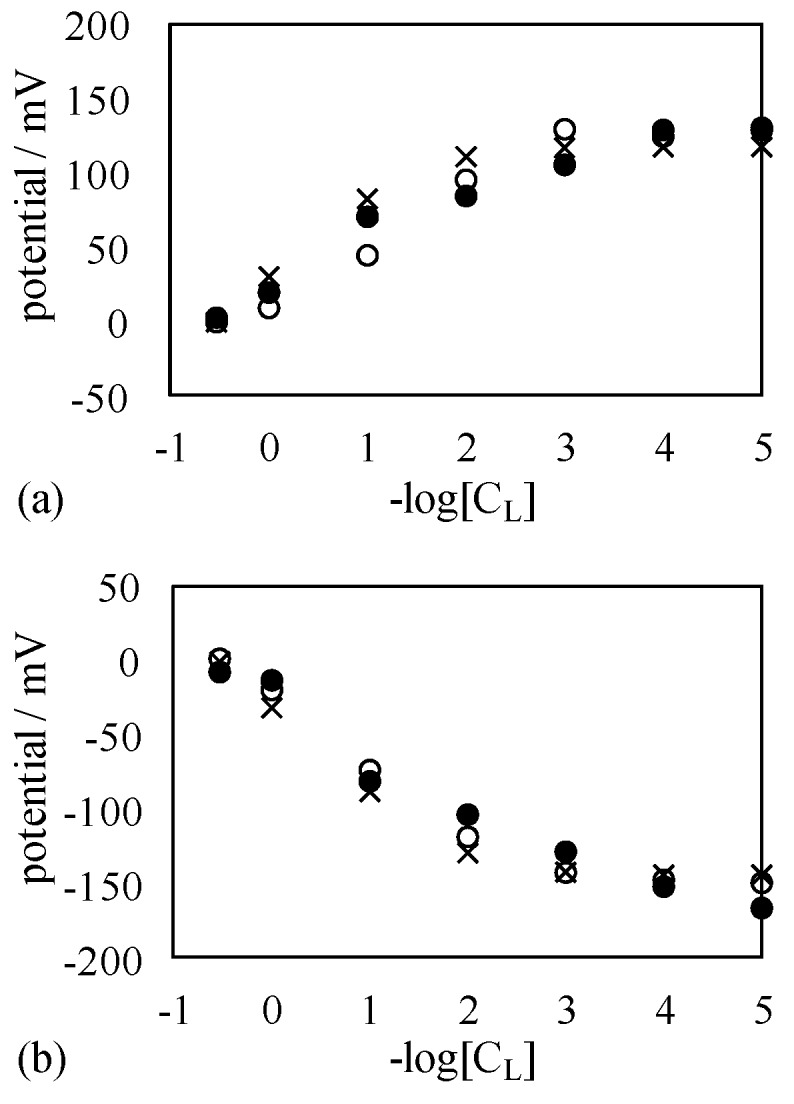
(**a**) Membrane potential shown in Figure 5a (○ mark) and Figure 7a (● mark) and the computed membrane potential based on the GHK equation assuming P_K_ = 1 and P_Cl_ = 0.008 (× mark); (**b**) Membrane potential shown in Figure 5b (○ mark) and Figure 7b (● mark) and the computed membrane potential based on the GHK equation assuming P_K_ = 0.008 and P_Cl_ = 1 (× mark).

The potential behaviors in Exp. I and II. Exp I are discussed and explained using a proposed model. Experimental results shown in Figure 5 and Figure 7 are all membrane potential across an impermeable membrane. According to the GHK equation, the nonzero membrane potential is generated only across the semi-permeable membrane, since the GHK equation states that the membrane potential is determined by the concentration of individual ions in the electrolytic solutions and the membrane permeability to those ions [1,7]. Without the membrane permeability to ions, the GHK equation collapses and it implies that the nonzero membrane potential cannot be generated. However, the nonzero potential was undoubtedly induced as shown in Figure 5 and Figure 7. Figure 10 shows the experimentally measured membrane potentials shown in Figure 5 and Figure 7 and the membrane potentials computed by employing GHK equation by appropriately choosing the membrane permeability of P_K_ and P_Cl_, where the GHK equation employed is given by Equation (1). Concerning Equation (1), [K^+^]_L_ and [K^+^]_R_ represent K^+^ concentration in the left and right solutions, respectively. [Cl^−^]_L_ and [Cl^−^]_R_ represent Cl^−^ concentration in the left and right solutions, respectively. P_K_ and P_Cl_ represent the membrane permeability to K^+^ and Cl^−^, respectively. Data shown with an “×” mark represents the computed membrane potential. Figure 10 indicates that appropriately choosing membrane permeability P_K_ and P_Cl_ can reproduce the experimentally obtained membrane potential, even though the membrane actually used for the experiment is impermeable to ions. Some scientists acknowledged that the quantity of permeability of P does not necessarily mean the actual membrane permeability to ion [29], or the quantity of permeability of P is not necessarily constant but is dependent on ion concentration [30], but such a treatment for theoretical evaluation of membrane potential is sort of makeshift evaluation for obtaining the desired theoretical membrane potential without any reasonable justification. Therefore, the authors doubt the validity of GHK equation. The authors thus believe the GHK equation is a wrong concept. Now, they need to find out a theory alternative to the GHK equation.

(1) $$\Delta V=-\frac{\text{RT}}{F}\ln\frac{P_{K}[K^{+}{]}_{L}+P_{\text{Cl}}[{\text{Cl}}^{-}{]}_{R}}{P_{K}[K^{+}{]}_{R}+P_{\text{Cl}}[{\text{Cl}}^{-}{]}_{L}}$$

One of the authors of this paper, Tamagawa, has studied the membrane potential across the impermeable membrane for the past 10 years. He reached the conclusion that the GHK equation is a wrong concept and the adsorption theory advocated by G. Ling is the right theory as the membrane potential generation mechanism [1,7,21,22]. As described in the introduction, Ling has emphasized that the membrane potential of cell is basically caused by the adsorption of mobile ions onto the adsorption sites of a cell. The authors would like to propose a mechanism about this nonzero membrane potential generation across the impermeable membrane by employing the Ling’s adsorption theory.

**Figure 11:**
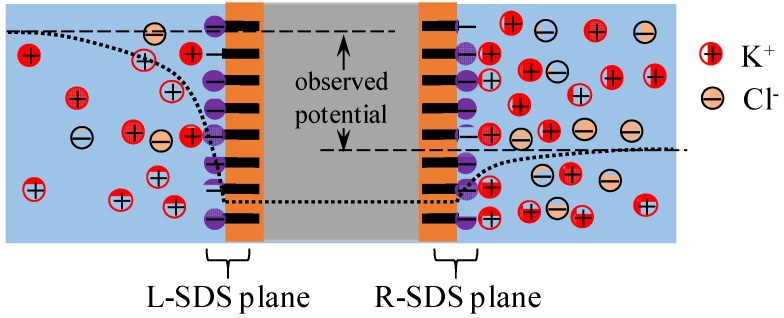
Ion distribution of two electrolytic solutions separated by a SMG90.

First of all, the membrane potential behavior shown in Figure 5a is explained by employing the Ling’s adsorption theory. The adsorption theory states that the adsorption of mobile ions in the electrolytic solution on the membrane surface causes the nonzero membrane potential [1,7]. Figure 11 shows the ion distribution in the two electrolytic solutions separated by SMG90, where the KCl concentration in the right solution is assumed to be higher than that in the left solution in this illustration. Since K^+^ concentration in the right solution is quite high, most of the negative charge of the SDS plane (hereafter called R-SDS plane) at the interface between SMG90 and the right KCl solution is neutralized by the adsorption of K^+^ (see Figure 11). On the other hand, most of the negative charge of the SDS plane (hereafter called R-SDS plane) at the interface between SMG90 and the left KCl solution is not neutralized, since K^+^ concentration in the left solution is low (see Figure 11).

The mobile ions in the right solution distributes in accordance with the Boltzmann distribution under the influence of slightly negatively charged R-SDS plane, while the mobile ions in the left solution distributes in accordance with the Boltzmann distribution under the influence of greatly negatively charged L-SDS plane. Therefore, the potential profile must be given as drawn in Figure 11 with dotted line. Membrane potential is experimentally measured using two electrodes. Those two electrodes are inserted into the bulk phases of left and right solutions, respectively. Hence, the potential measured is indicated by “observe potential” in Figure 11, where the potential detected by the electrode in the right solution is the reference potential of 0 mV. As long as the K^+^ concentration in the left solution is lower than that in the right solution, the observed potential is expected to be positive, since the mobile ions distribute in accordance with Boltzmann distribution under the influence of greatly negatively charged L-SDS plane and slightly negatively charged R-SDS plane. In fact, the potential experimentally observed was basically positive as shown in Figure 5a. The more negative the charge density of L-SDS plane becomes, the higher the potential observed is expected to be. The experimental result is in line with the expectation as shown in Figure 5a. Just opposite phenomenon is induced, when CMG90 was used in place of SMG90 as shown in Figure 5b, since the head charge of CTAB is positive.

Ling’s adsorption theory suggests that the entire structure of membrane is not the primary factor in the nonzero membrane potential generation. Ling’s adsorption theory suggests that the mobile ion adsorption on the membrane adsorption sites plays a central role for the induction of nonzero membrane potential, *i.e.*, even though the differently structured membranes are used for separating two electrolytic solutions, the membrane potentials observed across those membranes must be the same, as long as the structure of adsorption sites on those membranes are same each other. The surface structure of SKBMG90 was same as that of SMG90 (see Figure 4 and Figure 6), although the entire structure of SKBMG90 was totally different from that of SMG90. As expected, following the Ling’s adsorption theory, the actual potential behavior across the SKBMG90 was virtually same as that across the SMG90 as shown in Figure 10a. This concept is also true for the potentials across the CKBMG90 and CMG90 as shown in Figure 10b. However, one may still argue that the GHK equation is applicable only to the system employing a semipermeable membrane not an impermeable membrane (SKBMG90 and CKBMG90 are completely impermeable), hence, the argument so far described is meaningless from the beginning. However, Tamagawa and Morita already clarified that point in their previous reports [21,22]. They found that the actual potential between two KCl solutions separated by the impermeable membrane was virtually same as the experimentally measured potential separated by the semipermeable membrane. They confirmed that the latter potential (across the semipermeable membrane) was quantitatively well reproducible by the GHK equation, and this confirmation suggests that even the former potential (across the impermeable) is reproducible by the GHK equation as well. This consequence made us wonder if the ion permeation through the membrane was the primary cause of membrane potential generation. Instead, Tamagawa and Morita proposed an alternative mechanism of potential generation across the impermeable material as well as the semipermeable membrane [21,22]. The mechanism proposed was based on the Ling’s adsorption theory [1,7], and it is basically the same theory explained above using Figure 11, *i.e.*, the ion adsorption on the membrane is induced, even when the membrane is not impermeable but semipermeable. Consequently, it results in the nonzero membrane potential generation. This mechanism was in harmony with the potential behaviors for both impermeable and permeable membrane cases.

To sum up, there is not a firm reason to justify the GHK equation which states that the membrane potential is generated by the ion permeation through the semipermeable membrane. Nonzero potential is generated even across the impermeable membrane, and even the membrane potential across the semipermeable membrane is explicable by the theory based on the Ling’s adsorption theory instead of GHK equation [1,7]. We do not have a decisive reason to rule out Ling’s adsorption theory as a membrane potential generation mechanism at all.

### 4.3. Mechanism of Potential Oscillation

**Figure 12:**
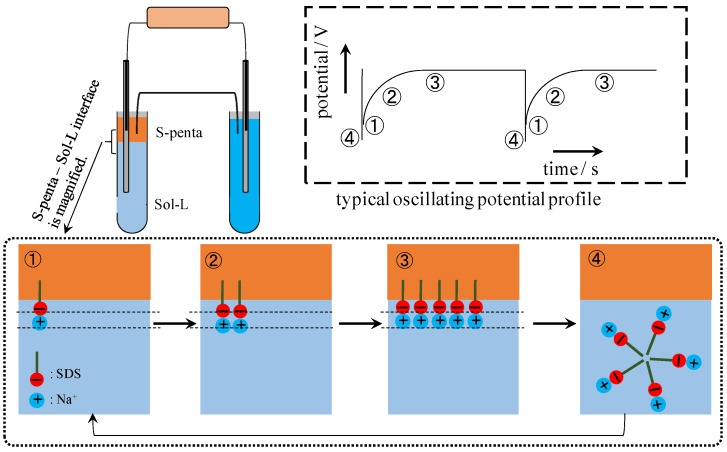
Typical potential profile between 0.3 M NaCl and 0.5 M KCl solutions separated by a silver wire & S-penta is illustrated in the dashed square and the molecular level mechanism of potential oscillation induced at the interface between S-septa and Sol-L is illustrated in dotted square.

Ling’s adsorption theory can explain the potential oscillation across the impermeable membrane shown in Figure 8 and Figure 9 as well [1,7]. Typical profile of oscillating potential generated between 0.3 M NaCl and 0.5 M KCl solutions separated by a silver wire & S-penta is illustrated in the dashed square in Figure 12, and this oscillating potential profile is analyzed here.

It is speculated that the potential oscillation is induced at the interface between S-penta and Sol-L. Therefore, molecular level electrochemical phenomenon at the interface is discussed. Once S-penta and Sol-L comes into contact with each other, SDS molecules tend to form a monolayer at the interface between them, and K^+^ in Sol-L are adsorbed on the negative head charge of SDS molecule as illustrated in ① in the dotted square in Figure 12, *i.e.*, a capacitor is formed at the state ①. Therefore, the potential slightly increases as indicated by ① in the potential profile in the dashed square in Figure 12. As time goes by, SDS molecules come to form a more densely charged capacitor as illustrated in the dotted square in Figure 12, the state ① → the state ② → the state ③. Consequently, the potential increases as indicated by ① → ② → ③ in the potential profile in the dashed square in Figure 12. SDS concentration at the interface between S-penta and Sol-L increased further as time went by, and once SDS concentration reached critical micelle concentration (CMC), the capacitor collapsed and a micelle formed as illustrated in the state ④ in the dotted square in Figure 12. At the very same moment when the capacitor collapses, the potential abruptly plunged as indicated by ④ in the potential profile shown in Figure 12. Since this process (the state ① through ④) repeated, the potential oscillation was observed. It is speculated that the potential oscillation, when C-penta was used, is induced basically by the same mechanism. Since the sign of head charge of CTAB contained in C-penta is just opposite to the sign of head charge of SDS, the potential peaks went positive going, while the potential peaks went negative going, when S-penta was used.

Potential oscillation mechanism we proposed is basically same as the widely and currently accepted mechanism [38,39,40,41,42,43,44,45,46,47,48,49]. It suggests that the ion transfer between two aqueous solutions across the membrane is not needed at all for the generation of oscillating potential (action potential-like potential), and potential oscillation is caused merely by the mobile ion adsorption-desorption process occurring at the surface of surfactant monolayer, and that mechanism is basically within the concept of Ling’s adsorption theory [1,7]. In the electrochemistry research field, people involved in the investigation on the potential oscillation phenomena have employed the fundamentally same theory as Ling’s adsorption theory without knowing it.

From the standpoint of nonlinear dynamics, Yoshikawa *et al.* proposed a mathematical model of the potential oscillation, which was induced between two electrolytic solutions separated by oil membrane [41]. They emphasized that the repetition of surfactant monolayer formation-destruction process at the interface between electrolytic solution and oil membrane caused the potential oscillation. Their theory was mathematics- and physics-based rigid theory. Although they employed some assumptions for building the mathematical model such that van der Waals equation for the surface pressure *vs.* surfactant concentration was expressed by – Z + Z^3^ (Z: surfactant concentration), those assumptions are quite plausible from the standpoint of chemistry and physical chemistry. Although their theory did not offer the molecular level mechanism of how the repetition of surfactant monolayer formation-destruction process induced the nonzero potential, it is quite reasonable theory, as long as the repetition of surfactant monolayer formation-destruction process can be interpreted as the repetition of capacitor formation-destruction process as illustrated in Figure 12. Hence, the molecular level model in Figure 12, which is based on the Ling’s adsorption theory, is quite plausible from the standpoint of mathematics, physics, chemistry and physical chemistry.

We would like to raise a question again. Why do we have to use the GHK equation instead of Ling’s adsorption theory in physiology, despite the fact that the successful outcomes have been realized in the electrochemistry research field by the fundamentally same theory as the Ling’s adsorption theory as so far described? What is the primary reason to attach more importance to the GHK equation than to the Ling’s adsorption theory in physiology research field? As described in at the end of Section 4.2, we do not have a decisive reason to rule out the Ling’s adsorption theory as a membrane potential generation mechanism at all. It is necessary to put forth a firm explanation to rule out the Ling’s adsorption theory, if someone would like to fully justify GHK equation: We need scientific reasons why the Ling’s adsorption theory is not that suitable as a membrane potential generation mechanism or why we have to use distinct two theories—GHK equation and another theory fundamentally same as Ling’s adsorption theory—in accordance with the research field physiology or electrochemistry. We believe that Ling’s adsorption theory is a simpler, more comprehensive and more suitable theory than GHK equation for explaining membrane potential behavior. Even though still people think that the GHK equation is a valid theory, we at least need to find a reason to rule out the Ling’s adsorption theory.

## 5. Conclusions

Membrane potential can even be generated across the impermeable membrane and action potential-like potential can be induced in nonliving system containing no ion channels or pumps. The membrane potential generation including the induction of action potential does not appear to be a phenomenon particular to the living system only. Without living or biological matters, such a phenomenon can be induced. The authors of this paper disagree with the current theory of membrane potential generation mechanism. The most simple and plausible explanation of those observations is the Ling’s adsorption theory. Electrical properties of cell are explicable without the functionalities of ion channels and/or pumps. Therefore, it is not fully nonsensical to speculate that the electrical properties of living system are governed by the same mechanism as what governs the electrical properties of nonliving system. Ling’s adsorption theory is a quite comprehensive theory and, it is applicable to both living and nonliving systems as an explanation of their electrical behavior.

People might argue that because Ling’s adsorption theory can explain the membrane potential behavior, it does not follow that the GHK equation is doubtful. However, the reverse is also true. *i.e.*, because GHK equation can explain the membrane potential behavior, it does not follow that the Ling’s adsorption theory is doubtful.

Ling’s adsorption theory attributes the membrane potential generation to the mobile ion adsorption on the adsorption sites, and undoubtedly the mobile ion adsorption on the adsorption site of membrane takes place whether the membrane is permeable or not. Therefore, it is necessary at least to find a mechanism of eliminating the potential generated by ion adsorption on the adsorption sites of “semipermeable” membrane, as long as they believe the full validity of GHK equation.

The readers of this paper might feel more or less uneasy with the discussion described in this paper, since the authors did not show any explicit mathematical expression of Ling’s adsorption theory. However, the authors are now in the middle of more comprehensive and quantitative study of Ling’s adsorption theory by developing the mathematical formulas of Ling’s adsorption. Hopefully, we can publish it within a few months.
